# For-Profit Program for All Inclusive Care for the Elderly Plans and Patient Characteristics

**DOI:** 10.1001/jamanetworkopen.2025.56296

**Published:** 2026-01-28

**Authors:** Katherine E. M. Miller, David J. Meyers

**Affiliations:** 1 Department of Health Policy and Management, Bloomberg School of Public Health, Johns Hopkins University, Baltimore, Maryland; 2 VA Partnered Evidence Based Resource Center, Boston Health Care System, Boston, Massachusetts; 3 Department of Health Services, Policy, and Practice, Brown University School of Public Health, Providence, Rhode Island

## Abstract

This cohort study describes characteristics and health care use patterns of Medicare enrollees in Program for All Inclusive Care for the Elderly (PACE) plans by ownership type.

## Introduction

The Program for All Inclusive Care for the Elderly (PACE) is an integrated care model for Medicare beneficiaries who qualify for nursing home–level care but wish to remain in the community.^1^ Historically nonprofit entities, PACE programs receive capitated Medicare and Medicaid payments to provide comprehensive medical and social services to participants through adult day centers to help beneficiaries age in the community by preventing hospitalizations and long-term nursing home placement.^1^ Approximately, 188 PACE programs exist nationally, with more than 82 000 participants.

Seminal evidence shows that PACE reduces hospital use.^1^ In 2016, regulatory changes allowed for-profit programs to enter the market, including private equity (PE)–backed firms.^2^ Evidence from other health care industries suggests that for-profit and PE entities can impact patient selection and pattens of service use to maximize profits.^3^ However, little evidence examines how PACE populations and their health care use patterns adapted after these market changes.^4^ Thus, we describe Medicare enrollee characteristics and health care use patterns in PACE by ownership type (nonprofit, for-profit, for-profit with PE, and for-profit without PE).

## Methods

This cohort study conformed to the STROBE reporting guidelines for cohort studies, and we completed analyses from May to November 2025. The Johns Hopkins Institutional Review Board approved the study. Our primary sources of data are 2021 Medicare and Medicaid claims data. We used the Medicare Master Beneficiary Summary File to identify and describe PACE enrollees, health care use, and mortality by linking plan identification numbers to a previously constructed dataset of PACE ownership characteristics.^5^ We incorporated Medicaid claims to identify nursing home use.

Our primary outcomes include emergency department (ED), inpatient, and skilled nursing facility use. Our primary exposures were (1) nonprofit vs for-profit status of PACE and (2) for-profit with vs without PE backing. We compared outcomes using χ^2^ tests for categorical variables and analysis of variance for continuous variables across groups using Stata MP-Parallel Edition version 17.0 (StataCorp). We reported 2-sided *P* values, with *P* < .05 indicating statistical significance.

## Results

Among 63 741 PACE enrollees in 2021, the mean (SD) age was 76.94 (9.83) years, and most enrollees were female (42 254 enrollees [66.3%]) and enrolled in Medicaid and Medicare (63 125 enrollees [99.0%]). In total, 50 184 individuals (78.7%) were served by nonprofit PACE, and 13 557 (21.3%) by for-profit PACE (9474 [14.9%] with PE and 4083 [6.4%] without PE backing). Among for-profit enrollees, 9474 (69.9%) were enrolled in a PE-backed PACE (Table).

**Table.** Descriptive Statistics by PACE Plan Ownership Status

Characteristic	PACE enrollees, No. (%)					*P* value	
	Total (N = 63 741 [100.0%])	Nonprofit total (n = 50 184 [78.7%])	For-profit total (n = 13 557 [21.3%])	For-profit and PE (n = 9474 [14.9%])	For-profit and no PE (n = 4083 [6.4%])	Nonprofit vs for-profit PACE	For-profit PACE with vs without PE
Demographics							
Age, mean (SD), y	76.94 (9.83)	77.29 (9.87)	75.66 (9.59)	76.02 (9.59)	74.82 (9.54)	<.001	<.001
Sex							
Male	21 487 (33.7)	16 594 (33.1)	4893 (36.1)	3351 (35.4)	1542 (37.8)	<.001	.008
Female	42 254 (66.3)	33 590 (66.9)	8664 (63.9)	6123 (64.6)	2541 (62.2)		
Race and ethnicity^a^							
American Indian or Alaska Native	308 (0.5)	259 (0.5)	49 (0.4)	34 (0.4)	15 (0.4)	<.001	<.001
Asian or Pacific Islander	3994 (6.3)	3230 (6.4)	764 (5.6)	275 (2.9)	489 (12.0)		
Black (or African-American)	12 152 (19.1)	9892 (19.7)	2260 (16.7)	1836 (19.4)	424 (10.4)		
Non-Hispanic White	34 552 (54.2)	26 302 (52.4)	8250 (60.9)	5533 (58.4)	2717 (66.5)		
Hispanic	11 681 (18.3)	9594 (19.1)	2087 (15.4)	1697 (17.9)	390 (10.0)		
Other	460 (0.7)	379 (0.8)	81 (0.6)	50 (0.5)	31 (0.8)		
Unknown	594 (0.9)	528 (1.1)	66 (0.5)	49 (0.5)	17 (0.4)		
Enrolled in Medicaid	63 125 (99.0)	49 761 (99.2)	13 364 (98.6)	9324 (98.4)	4040 (98.9)	<.001	.02
Health care use							
Any ED use	3185 (5.0)	2445 (4.9)	740 (5.5)	525 (5.5)	215 (5.3)	.005	.52
No. of ED visits, mean (SD)	2.08 (2.41)	2.02 (2.24)	2.27 (2.87)	2.30 (2.95)	2.20 (2.67)	.03	.67
Any inpatient stays	1984 (3.1)	1557 (3.1)	427 (3.1)	291 (3.1)	136 (3.3)	.78	.43
Any skilled nursing facility stays	1275 (2.0)	1003 (2.0)	272 (2.0)	206 (2.2)	66 (1.6)	.95	.03
Any long-term nursing home stays	4457 (7.0)	3779 (7.5)	678 (5.0)	513 (5.4)	165 (4.0)	<.001	<.001
Program characteristics							
PACE disenrollment^b^	4221 (6.6)	3159 (6.3)	1062 (7.8)	735 (7.8)	327 (8.0)	<.001	.65
Mortality	6391 (10.0)	5118 (10.2)	1273 (9.4)	898 (9.5)	375 (9.2)	.005	.59
PE backing	9474 (14.9)	0	9474 (69.9)	9474 (100.0)	0	NA	NA

Abbreviations: ED, emergency department; NA, not applicable; PACE, Program for All Inclusive Care for the Elderly; PE, private equity.

^a^ We use the Research Triangle Institute race code, which is an algorithm that builds upon the Medicare self-reported race and ethnicity of each beneficiary by also including data from the Social Security Administration data to better capture more beneficiaries as Hispanic or Asian. The category of other captures any other race not otherwise specified.

^b^ We exclude individuals who died from the denominator when calculating the percentage of individuals who disenroll from PACE.

We observed differences in enrollee characteristics by ownership type. Compared with nonprofit PACE enrollees, for-profit enrollees were younger and more likely to be male, non-Hispanic White, have any ED visit, have a greater number of ED visits, and disenroll from PACE. Compared with nonprofit PACE enrollees, a lower share of for-profit PACE enrollees were enrolled in Medicaid, experienced a long-stay nursing home visit, and died. Among for-profit enrollees, those in PE-backed programs had more skilled and long-term nursing home stays.

## Discussion

This cohort study found that although most PACE enrollees were served by nonprofit PACE programs, for-profit program enrollees were more likely to use the ED and disenroll from PACE, have a greater number of ED visits, and were less likely to die. Results are more pronounced when comparing nonprofit enrollees with enrollees in PE PACE. These findings have implications for policies addressing the access of PACE and monitoring the quality of PACE programs. While privatization and corporatization could increase access to PACE,^6^ it may also result in firms strategically choosing healthier patients and/or reducing services to increase profits.

Limitations include that we used cross-sectional data and are unable to make causal inferences, and we cannot capture long-term nursing home stays. Examining differences within younger enrollee cohorts is an important next step to disentangle the mechanisms driving results (eg, cohort effects vs ownership status or the combination thereof). Given the heterogeneity in patient characteristics and outcomes, further research is needed to understand the causal impacts on patients to ensure equitable access to high-quality PACE programs.

## Notes

Supplement. Data Sharing Statement
