# Supplementary material for: For-Profit Program for All Inclusive Care for the Elderly Plans and Patient Characteristics
Source: JAMA Netw Open. 2026 Jan 28;9(1):e2556296. doi: 10.1001/jamanetworkopen.2025.56296 (PMC12853201; doi:10.1001/jamanetworkopen.2025.56296)
Supplement: Supplement. — Data Sharing Statement [file jamanetwopen-e2556296-s001.pdf]

Data Sharing Statement

Miller. For-Profit Program for All Inclusive Care for the Elderly Plans and Patient Characteristics. *JAMA Netw Open*. Published January 28, 2026.
doi:10.1001/jamanetworkopen.2025.56296

Data
Data available: No

Additional Information
Explanation for why data not available: We are not able to make our data publicly available due to the data use agreement with the Centers for Medicare & Medicaid Services.
